# Trajectories of family resilience among caregivers of older adults with dementia: a longitudinal study in community-based geriatric care settings

**DOI:** 10.3389/fpubh.2026.1809509

**Published:** 2026-05-13

**Authors:** Jialin Lv, Mengyu Yang, Jia Mao, Jiawen Pan, Qingxin Gu, Lixiu Zhang

**Affiliations:** 1 Department of Nursing, College of Medical Science, Huzhou University, Zhejiang, China; 2 Affiliated Cixi Hospital, Wenzhou Medical University, Zhejiang, China

**Keywords:** decision tree, dementia caregivers, family resilience, geriatric care, logistic regression, older adults, predictive factors, trajectory analysis

## Abstract

**Background:**

Dementia poses significant challenges for family caregivers, yet little is known about the developmental trajectories of family resilience and their predictors in this population. Understanding these patterns is essential for developing targeted interventions to support caregivers and enhance care quality. This study aims to identify and predict trajectories of family resilience among dementia caregivers using complementary logistic regression and decision tree approaches, and to explore key factors influencing these trajectories.

**Methods:**

A three-wave longitudinal study was conducted among 239 dementia caregivers from 19 communities in Huzhou, China, between June 2023 and July 2024. Growth Mixture Modeling (*GMM*) was applied to trajectories. Binary logistic regression and decision tree models were employed to analyze predictors of these trajectories, and Receiver Operating Characteristic (*ROC*) curves were used to evaluate model performance.

**Results:**

Two distinct family resilience trajectories were identified: a “low resilience-rapidly declining” group and a “high resilience-slowly rising” group. Key predictors included caregiver empowerment, self-efficacy, self-rated health, dementia knowledge, social support, and relationship with the patient. The logistic regression model demonstrated higher sensitivity, whereas the decision tree model showed higher specificity. The complementary use of both models enhanced predictive accuracy and interpretability.

**Conclusion:**

Family resilience among Chinese dementia caregivers follows heterogeneous trajectories primarily predicted by caregiver empowerment and self-efficacy. The identified thresholds offer candidate criteria for early identification of high-risk families, pending external validation. These findings support the development of tiered, empowerment-focused interventions within community-based dementia care systems.

## Graphical abstract

**Figure:**
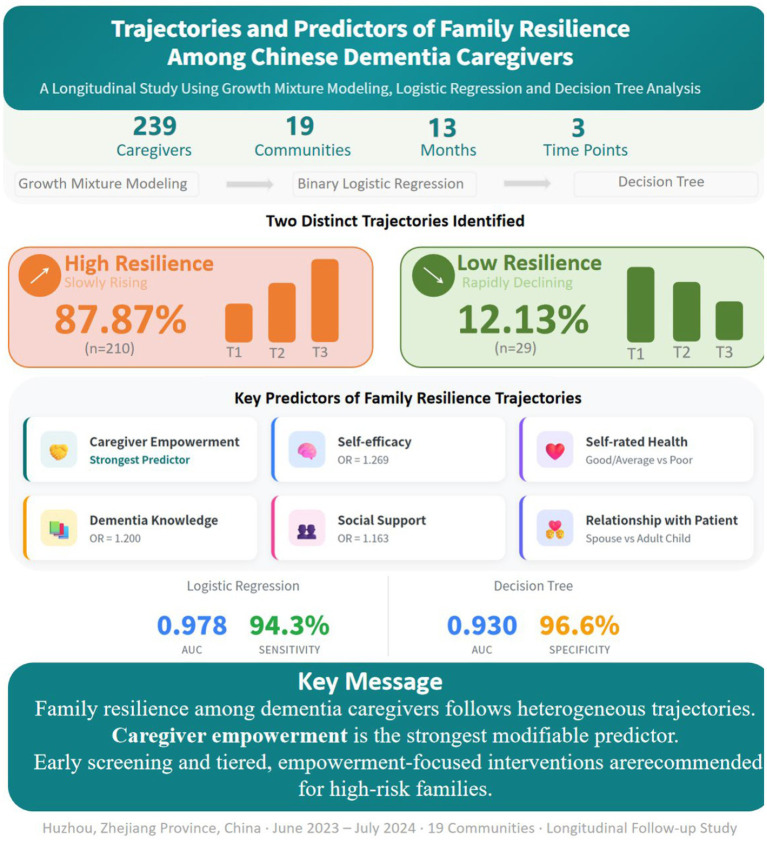

## 1 Background

The rapid growth of the geriatric population worldwide has placed unprecedented pressure on healthcare systems, particularly geriatric care services, with this phenomenon being especially pronounced in China (1). Against this backdrop, dementia has emerged as one of the most significant public health challenges of our time (2). Dementia, also referred to as major neurocognitive disorder, is a clinical syndrome characterized by progressive deterioration of cognitive function, often accompanied by behavioral and psychological symptoms (BPSD) (3). These cognitive and behavioral changes lead to declining abilities in activities of daily living (ADL), instrumental activities of daily living (IADL), and broader social functioning (4). As the condition progresses, individuals with dementia require increasing levels of care, imposing substantial physical, emotional, and financial burdens on their caregivers (5). Globally, as of 2023, over 11 million family members and unpaid caregivers collectively provided an estimated 18.4 billion hours of care to individuals living with dementia (6). In China, influenced by Confucian values of filial piety and traditional family-centered culture, the majority of individuals with dementia are cared for at home by family members. Adult children account for more than half of all primary caregivers in China (7, 8), a proportion substantially higher than that reported in Western countries (9). While this cultural practice reflects strong family bonds, prolonged caregiving often results in considerable psychological distress, physical exhaustion, and disruptions to family functioning (10, 11). Such burdens are further compounded in the Chinese cultural context, where Confucian norms of filial piety simultaneously motivate intensive family-based caregiving and constrain help-seeking behaviors, creating unique pressures on family resilience. Notably, the act of caring for people with dementia has been shown to have repercussions on the caregiver’s own cognitive and physical health, contributing to deterioration in informal caregiver well-being and performance. This dynamic necessitates the development of culturally tailored preventive programs targeting the Chinese population (12). Given the significant physical, emotional, and financial burdens on caregivers, understanding how families adapt and maintain resilience has become a critical research priority.

Family resilience, also referred to as family elasticity, describes the capacity of a family to adapt flexibly to stressful or adverse circumstances, thereby demonstrating its ability to recover and bounce back. This ability enables the restoration of normal functioning, helps maintain overall family coherence, and facilitates recovery from distress, ultimately leading to enhanced resilience and improved family performance (13, 14). Studies indicate that enhancing family resilience can reduce caregiver burden, facilitate family adaptation, and play a significant role in conserving public medical resources and alleviating the societal burden of disease (15, 16).

In recent years, the field of family resilience research has been expanding, and significant progress has been made in the construction of theoretical frameworks and the development of intervention strategies (17, 18); however, despite these advances, the evidence base regarding the developmental trajectories of family resilience among caregivers of people with dementia remains in its exploratory stages (14, 19). First, existing research on predictors of resilience has primarily focused on cross-sectional assessments of resilience levels, capturing only static snapshots rather than examining the evolution of resilience over time (14). Although some studies have identified individual-level factors, such as caregiver burden, perceived social support, and coping strategies, as correlates of resilience at specific time points, it remains unclear whether and how these factors predict different longitudinal trajectories. Second, existing research has confirmed that family resilience is a dynamically evolving concept: Arielle (19) demonstrated that it fluctuates with changes in levels of hope and distress. At the same time, another study found that family resilience shifts in response to family adaptation and phased interventions (20). This dynamism implies that trajectory-level predictions require analytical perspectives distinct from those used in static measurements, and such perspectives have not yet been systematically applied to this population. Furthermore, most relevant studies have been conducted in Western or non-dementia-specific contexts (14), and their findings may be difficult to directly apply to Chinese dementia caregivers, as their experiences are influenced by unique cultural values and different healthcare system structures.

To address these gaps, the present study adopted a longitudinal design and an innovative dual-model analytical approach. The specific objectives were threefold: (1) to identify distinct trajectories of family resilience among Chinese dementia caregivers using growth mixture modeling (GMM); (2) to examine predictors of these trajectories through both binary logistic regression and decision tree models; (3) to compare the predictive performance of these two complementary approaches. By integrating the strengths of both models-logistic regression for identifying independent predictors and decision tree for revealing hierarchical decision rules, this study aimed to provide a comprehensive understanding of the factors influencing family resilience trajectories, thereby informing the development of differentiated, evidence-based family support interventions for dementia caregivers. Among the limited studies in this area, the present study is one of the few to apply a dual-model approach combining logistic regression and decision tree analysis to identify and predict family resilience trajectories in dementia caregivers, representing a potentially valuable methodological contribution to this emerging field.

## 2 Methods

### 2.1 Theoretical foundation

This study was guided by the resilience-driven systems theory proposed by Zheng Min (21), which posits that individuals facing chronic stress mobilize three interacting categories of resources: personal, family, and social, to buffer risk, maintain functioning, and achieve physical and psychological equilibrium. This framework is particularly suitable for the context of dementia caregiving, where sustained caregiving demands require the simultaneous deployment of intrapersonal capacities, family-level caregiving conditions, and external support networks.

Within this framework, candidate predictors were selected according to four pre-specified criteria: (i) conceptual correspondence with one of the three resource domains defined by the model; (ii) prior empirical evidence linking the variable to family resilience or caregiver adaptation in dementia or other chronic-illness caregiving populations (14, 19, 22–24); (iii) measurability in the Chinese cultural context using instruments with established reliability and validity; and (iv) potential responsiveness to change over the study period, consistent with a trajectory-based analytical design.

**Figure 1:**
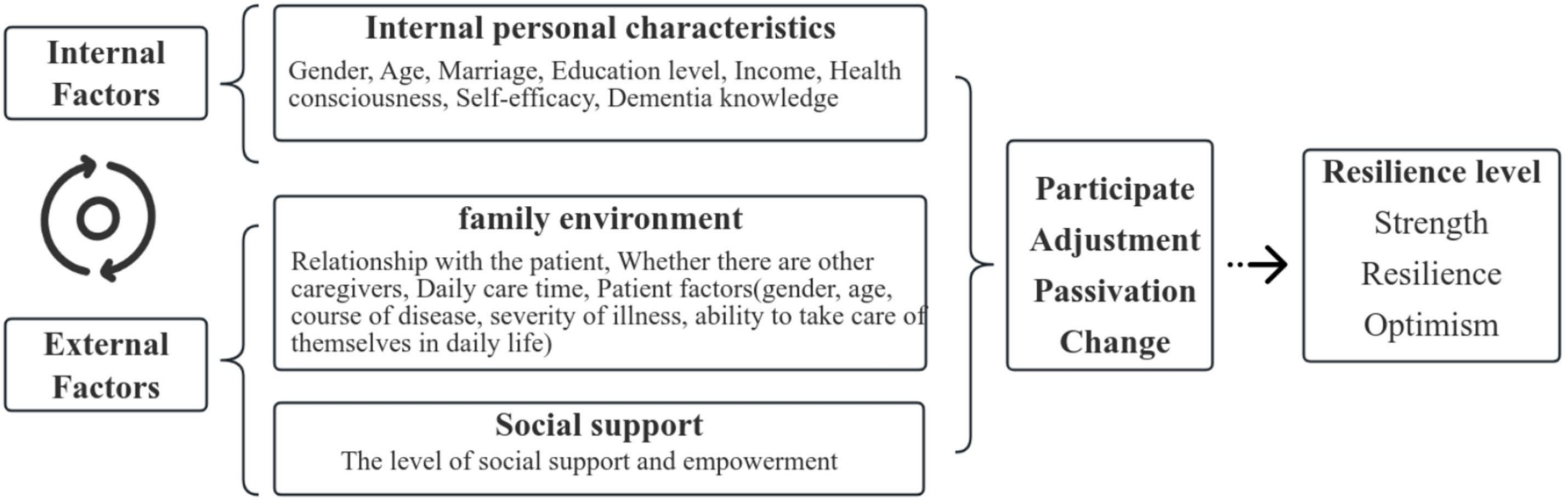
The relationship between various variables.

Based on these criteria, variables were mapped onto the three domains as follows. The personal domain included caregiver demographics (age, gender, education, relationship with the patient), self-rated health, general self-efficacy, dementia knowledge, and caregiver empowerment. The family domain included characteristics of the care recipient and the caregiving context (disease course, dementia severity, activities of daily living, daily care duration, and presence of other co-caregivers). The social domain was operationalized through the three dimensions of the Social Support Rating Scale (subjective support, objective support, and support utilization). Consistent with the resilience-driven systems model, we hypothesized that these three domains would jointly and interactively predict membership in distinct family resilience trajectories: greater personal resources, more manageable family caregiving demands, and stronger social support were each expected to increase the probability of belonging to the higher-resilience trajectory. The conceptual framework is illustrated in Figure 1.

### 2.2 Participants

This longitudinal study utilized a convenience sample of family caregivers of individuals with dementia residing in Huzhou City, Zhejiang Province, China. Participants were recruited from 19 community health service centers between May and June 2023. Recruitment was facilitated by community healthcare staff, who identified potentially eligible families through local health records and home-visit registries. Research assistants then contacted these caregivers via telephone or in person at the community centers to introduce the study, confirm eligibility, and obtain written informed consent prior to baseline data collection.

Inclusion criteria: (1) aged ≥18 years; (2) self-identified as the primary unpaid caregiver, defined as the family member who (a) had assumed the principal responsibility for the care recipient’s daily living assistance and care-related decision-making, and (b) had provided care for ≥3 months; when multiple family members shared caregiving, the one reporting the highest weekly caregiving hours was selected as the primary caregiver; (3) the care recipient had a documented diagnosis of dementia made by a neurologist, geriatrician, or psychiatrist at a secondary or tertiary hospital, based on DSM-5 criteria as recommended by the 2018 Chinese Guideline for Diagnosis and Treatment of Dementia and Cognitive Disorders (25), and the research team verified diagnoses through review of medical records provided by the family; (4) provided informed consent and volunteered to participate.

Exclusion criteria: (1) the caregiver had major physical diseases, malignant tumors, or mental illnesses; (2) the caregiver was a paid one, such as a nurse, nanny, or hired caregiver; (3) the caregiver was unable to communicate effectively.

Withdrawal criteria: (1) the caregiver requested withdrawal from the study for various reasons during the study survey; (2) the study was interrupted due to changes in the caregiver or the accidental death of the patient.

### 2.3 Sample size justification

Based on Kim’s simulation study on sample size requirements for growth mixture modeling, a minimum sample size of 200 was required (26). Considering 15% lost to follow-up, the minimum sample size is 236. Finally, 239 caregivers completed the follow-up survey in this study. While this total sample size meets the GMM requirement, it provides limited statistical power for detecting predictors of the smaller, low-resilience subgroup. The anticipated class imbalance may result in unstable estimates and wide confidence intervals in subsequent predictive models. This research protocol received approval from the Ethics Committee of Huzhou University (Approval number: 202401–17; Clinical trial number: not applicable).

### 2.4 Data collection procedures

Based on previous studies (22, 24, 27–29) and expert suggestions, the survey interval was set at 6 months. Three time points were selected as the minimum number required to identify linear and quadratic change patterns in Growth Mixture Modeling, providing an initial, parsimonious characterization of resilience trajectories. Three rounds of data collection were conducted: baseline (T1, June–July 2023), second (T2, December 2023–January 2024), and third (T3, June–July 2024).

At T1, the researcher, along with three trained members of the research team, conducted a face-to-face questionnaire survey at the community health center or in the homes of caregivers, with the assistance of staff at the community health service center. Before the survey, the researcher explained the purpose and significance of the study, and attention was paid to obtaining informed consent from caregivers during the filling process. The questionnaires were to be filled out by the caregivers themselves. For those who were unable to fill them out independently, the interviewer would explain the questions one by one and complete the questionnaires on their behalf, without leading the responses. The questionnaires were collected on the spot after being filled out.

At T2 and T3, face-to-face questionnaire surveys, as well as methods such as phone calls and WeChat, were used for follow-up data collection. After the survey was completed, the researcher immediately reviewed the quality of the questionnaire. If there were doubts about the responses, the researcher would personally confirm with the respondent.

Item-level missing data (occurring in < 5% of cases across all scales) were handled using multiple imputation by chained equations (MICE) with 20 imputed datasets. For the longitudinal GMM analysis, participants with at least one complete assessment (T1, T2, or T3) were included under the missing-at-random assumption.

### 2.5 Research tools

All instruments used in this study were psychometrically validated tools previously applied in Chinese populations, comprising either original Chinese scales or officially translated and culturally adapted versions of international instruments; full source references are cited alongside each scale below. Their dimensions, item counts, score ranges, and Cronbach’s *α* coefficients (both from original validation studies and from the present sample) are summarized in Table 1, with a brief description of each scale provided in the following subsections.

**Table 1:** Summary of measurement instruments.

Scale	Dimensions	Score range	Cronbach’s *α*	Reference
FRAS	Family communication, problem solving, social resource utilization, and maintaining a positive attitude (32 items)	32–128	0.960	Li et al. (30)
EFCD	Excellence in dementia care practice, understanding the nature of dementia care, taking care of oneself and the patient, peer support (16 items)	0–48	0.904	Sakanashi and Fujita (31); Xiao et al. (55)
Self-efficacy scale	General self-efficacy (10 items)	10–40	0.818	Wang et al. (32)
SSRS	Subjective support, objective support, utilization of support (10 items)	12–66	0.820	Xiao (33)
DKAS	Etiology, characteristics, communication & behavior, care precautions, risk and health promotion (25 items)	0–50	0.801–0.818	Annear et al. (56); Zhang (34)

Higher scores indicate better levels of the respective construct for all scales.

#### 2.5.1 General information questionnaire

It was self-designed by the researchers, including the gender, age, marital status, educational level, and relationship with the patient of the caregiver, as well as the gender, age, course of disease, and dementia severity of the person being cared for [refer to the 2018 Chinese Guideline for Diagnosis and Treatment of Dementia and Cognitive Disorders (25)].

#### 2.5.2 Chinese version of the family resilience assessment scale (FRAS)

This scale measures three dimensions (family communication and problem solving, social resource utilization, and maintaining a positive attitude). Total scores range from 32 to 128, with higher scores indicating higher family resilience (30).

#### 2.5.3 Empowerment scale for family caregivers of community-dwelling people with dementia (EFCD)

The EFCD captures caregivers’ empowerment across four aspects: excellence in dementia care practice, understanding the nature of dementia care, taking care of oneself and the patient, and peer support. Higher scores reflect stronger empowerment (24, 31). The test–retest reliability in this study was 0.822.

#### 2.5.4 Self-efficacy scale

This 10-item scale measures general self-efficacy. Total scores range from 10 to 40, with higher scores indicating stronger self-efficacy (32).

#### 2.5.5 Social support rating scale (SSRS)

The SSRS evaluates perceived social support through three dimensions: subjective support, objective support, and utilization of support. Higher scores denote stronger perceived social support (33).

#### 2.5.6 Dementia knowledge assessment scale (DKAS)

This scale includes four dimensions (etiology and characteristics, communication and behavior, care precautions, risk and health promotion). Total scores range from 0 to 50, with higher scores indicating greater dementia knowledge (34).

### 2.6 Data analysis and statistical methods

Statistical analysis was performed using R 4.5.1 and IBM SPSS Statistics 27.0. Continuous variables were assessed for normality using the Shapiro–Wilk test. Normally distributed continuous data are presented as mean and standard deviation (*SD*), and non-normally distributed continuous data are presented as median with interquartile range (IQR). Categorical variables are summarized as frequencies and percentages. For between-group comparisons, the independent samples *t-*test was used for normally distributed continuous variables, and the Mann–Whitney *U* test was used for non-normally distributed continuous variables. The chi-square ( χ2 ) test or Fisher’s exact test was used for categorical variables, as appropriate.

Firstly, the Growth Mixture Modeling (GMM) was constructed to identify potential heterogeneous trajectory categories of family resilience among caregivers of individuals with dementia. It provides two factors: intercept (*I*) and slope (*S*) to characterize developmental trajectories, measured by means and variance. The mean of the intercept factor represents the average initial state of the corresponding category group. At the same time, its variance describes the degree of dispersion between individuals in a particular time category. Models were fitted to determine the optimal number of potential categories, including Akaike Information Criterion (*AIC*), Bayesian Information Criterion (*BIC*), Adjusted Bayesian Information Criterion (*ABIC*), Entropy (*H*), Lo–Mendell–Rubin (*LMR*), and the Bootstrap-based Bootstrap Likelihood Ratio Test (*BLRT*). The lower the statistical values of *AIC*, *BIC*, and *ABIC*, the better the model fit. *H* is more than 0.8, indicating that the model’s classification accuracy is higher than 90%. When *LRT* and *BLRT* were statistically significant (*p* < 0.05), it was indicated that model K had more variance and a better fit than model K-1. Finally, these fitting criteria are combined, taking into account the simplicity and interpretability of the model, as well as its clinical practical significance, to determine the final number of trajectory categories.

Once the family resilience trajectories were determined, each caregiver was assigned to their most likely trajectory class based on their highest posterior probability (average posterior probability: 0.96). This “class assignment” approach was used for subsequent predictive modeling, and the potential predictors of different trajectories were preliminarily screened using one-way analysis, and variables with statistically significant differences in the univariate analysis were introduced into the further Binary Logistic Regression Model (*BLRM*). In addition, as an alternative model, the Chi-square Automatic Interaction Detection (*CHAID*) algorithm was used to construct a decision tree model, similar to *BLRM*; only variables with statistically significant differences were selected for the decision tree analysis. The maximum tree depth was set to 3 levels. To mitigate overfitting given the small size of the minority class, a minimum split size of 10 and a minimum child node size of 5 were implemented for the decision tree, and predictor variables were limited to a pre-specified set based on the theoretical framework. Due to the limited event and sample size, the minimum cases in the parent node and child node were set to 20 and 10, respectively. The Receiver Operating Characteristic (*ROC*) curve was used to visualize the model’s performance. The model’s performance was evaluated by calculating sensitivity, specificity, the Youden index, the Area Under the Curve (*AUC*), and its 95% confidence interval (*CI*). To assess model calibration, the Hosmer-Lemeshow goodness-of-fit test was employed for the logistic regression model, with a non-significant *p* > 0.05 indicating good calibration. For the decision tree model, calibration was visually assessed using a calibration plot comparing predicted versus observed probabilities across risk groups. A two-tailed *p* < 0.05 was considered statistically significant.

For the identification of resilience trajectories, the total resilience scores of 239 caregivers’ families were used as the indicator of observation. GMM was employed to analyze the fit, with the number of categories increased sequentially from 1 to 4 to determine the optimal model based on fit indices (*AIC*, *BIC*, *ABIC*, *LMR*, and *BLRT*) and clinical interpretability. Subsequently, potential predictors of different trajectory categories were preliminarily screened using a one-way analysis of variance. Only variables with statistically significant differences (*p* < 0.05) in the univariate analysis were introduced into the further *BLRM* and the *CHAID* decision tree model. In the logistic regression model, the family resilience trajectory category was set as the dependent variable (with the low resilience group as the reference).

## 3 Results

### 3.1 Baseline characteristics of participants

In this study, 246, 241, and 239 caregivers of T1, T2, and T3, respectively, completed the tracking survey. During this period, a total of 9 participants dropped out due to reasons such as loss to follow-up (4 cases), caregiver change (4 cases), and patient death (1 case). Therefore, the number of participants decreased from 246 to 239, resulting in a total loss rate of 2.85%. General information is shown in Table 2.

**Table 2:** Single-factor analysis of family resilience trajectories in dementia caregiving.

Variables	Total sample (*n* = 239)	Low resilience-rapidly declining group (*n* = 29)	High resilience-slowly rising group (*n* = 210)	Statistics	*p*
Caregiver information					
Age (year, mean ± SD)	55.64 ± 11.22	57.07 ± 11.88	55.45 ± 11.14	0.729	0.467
Gender [*n*(%)]				0.035	0.852
Male	111 (46.4)	13 (44.8)	98 (46.7)		
Female	128 (53.6)	16 (55.2)	112 (53.3)		
Situation of spouses [*n*(%)]				0.042	0.837
Have spouse	203 (84.9)	25 (86.2)	178 (84.8)		
No spouse	36 (15.1)	4 (13.8)	32 (15.2)		
Educational attainment [*n*(%)]				0.666	0.881
Primary and below	106 (44.4)	14 (48.3)	92 (43.8)		
Junior high school	76 (31.8)	8 (27.6)	68 (32.4)		
High school/secondary school	44 (18.4)	6 (20.7)	38 (18.1)		
College and above	13 (5.4)	1 (3.4)	12 (5.7)		
Monthly salary (Yuan) [*n*(%)]				1.399	0.692
<1,000	103 (43.1)	10 (34.5)	93 (44.3)		
1,000 ~ 2,999	103 (43.1)	15 (51.7)	88 (41.9)		
3,000 ~ 4,999	29 (12.1)	4 (13.8)	25 (11.9)		
>5,000	4 (1.7)	0 (0.0)	4 (1.9)		
Relationship with the patient [*n*(%)]				7.900	0.019
Spouse	57 (23.8)	5 (17.2)	52 (24.8)		
Others	45 (18.8)	11 (37.9)	34 (16.2)		
Kid	137 (57.3)	13 (44.8)	124 (59.0)		
Self-rated health [*n*(%)]				29.299	<0.001
Poor	62 (25.9)	19 (55.2)	43 (21.9)		
Average	119 (49.8)	8 (37.9)	111 (51.4)		
Good	58 (24.3)	2 (6.9)	56 (26.7)		
Other caregivers [*n*(%)]				0.001	0.976
Yes	123 (51.5)	15 (51.7)	108 (51.4)		
No	116 (49.5)	14 (48.3)	102 (48.6)		
Daily care duration [*n*(%)]				8.155	0.017
≥4 h and<8 h	51 (21.3)	2 (6.9)	49 (23.3)		
≥8 h and<12 h	87 (36.4)	8 (27.6)	79 (37.6)		
≥12 h	101 (42.3)	19 (65.5)	82 (39.0)		
Dementia knowledge (Mean ± SD)	22.47 ± 6.84	20.10 ± 5.88	22.80 ± 6.91	1.999	0.047
Social support (Mean ± SD)	31.97 ± 4.08	29.21 ± 4.65	32.35 ± 3.86	4.008	<0.001
Self-efficacy (Mean ± SD)	25.19 ± 6.29	22.45 ± 6.00	25.57 ± 6.25	2.532	0.012
caregiver empowerment (Mean ± SD)	30.11 ± 6.91	19.76 ± 6.00	31.54 ± 5.71	10.349	<0.001
Patient information					
Age (years, mean ± SD)	70.97 ± 9.24	68.72 ± 11.35	71.28 ± 8.90	1.400	0.163
Gender [*n*(%)]				0.934	0.334
Male	119 (49.8)	12 (41.4)	107 (51.0)		
Female	120 (50.2)	17 (58.6)	103 (49.0)		
Disease severity [*n*(%)]				14.175	0.001
Mild	86 (36.0)	5 (31.0)	81 (36.7)		
Moderate	120 (50.2)	13 (31.0)	107 (52.9)		
Severe	33 (13.8)	11 (37.9)	22 (10.5)		
Disease course [years, *M* (*P_25_*, P*_75_*)]	5.0 (3.0, 6.0)	6.0 (3.5, 10.5)	4.0(3.0, 6.0)	2.238	0.025
Activities of daily living [*n*(%)]				6.836	0.033
Mild	74 (31.0)	5 (17.2)	69 (32.9)		
Moderate	129 (54.0)	15 (51.9)	114 (54.3)		
Severe	36 (15.1)	9 (31.0)	27 (12.9)		

### 3.2 Identification and determination of resilience trajectories in families of caregivers of patients with dementia

**Figure 2:**
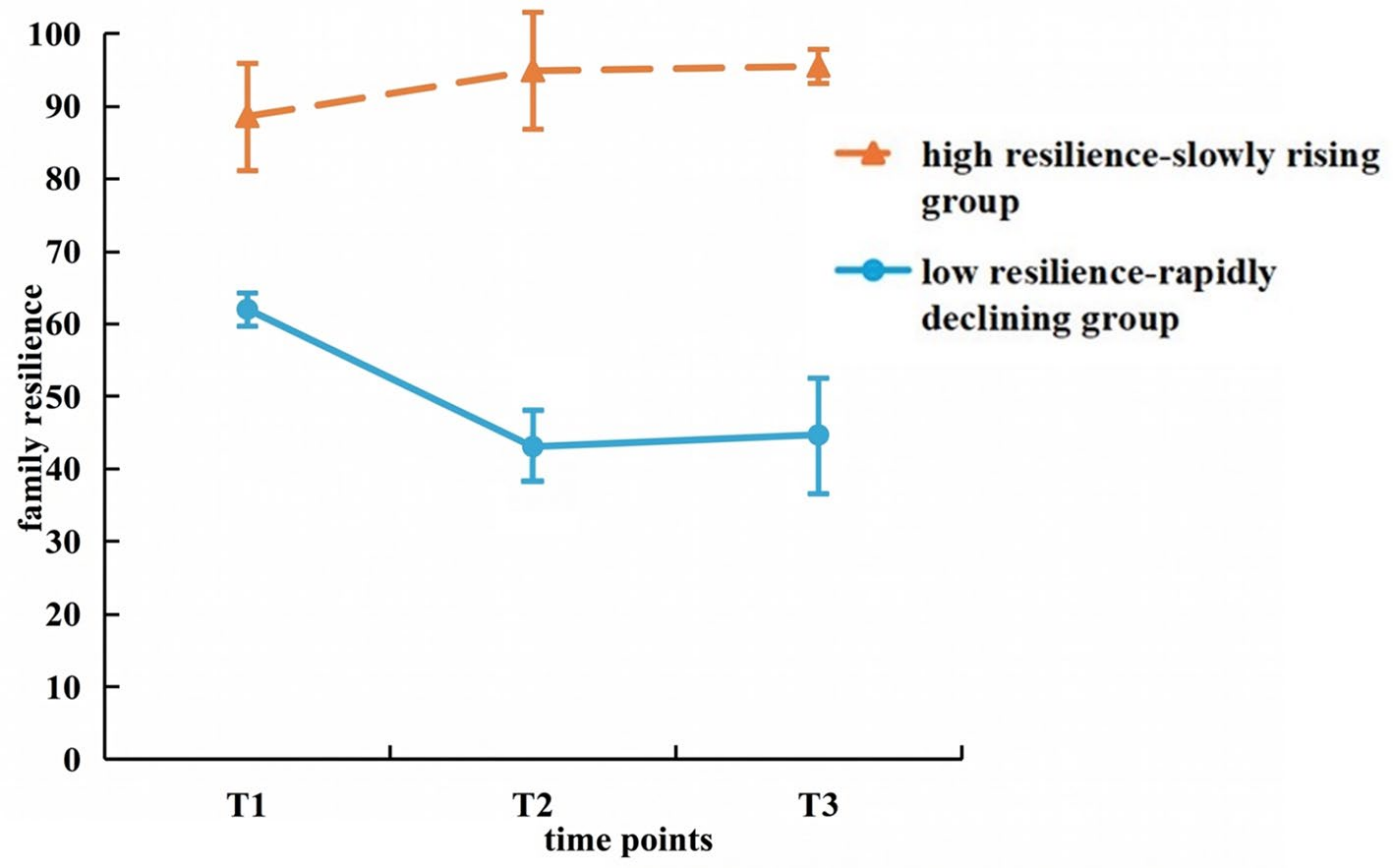
Trajectory of potential categories of family resilience for dementia caregivers. Plotted values represent observed group means at each time point. Error bars indicate the standard error (SE), representing the variability within each group. Model-estimated intercepts and slopes derived from GMM are reported in the text.

Based on the GMM analyses and model fitting criteria (Table 3), two distinct trajectories of family resilience were identified (Figure 2). Named according to their developmental characteristics, Category 1: this group of caregivers had an initial low level of family resilience (*I* = 47.690, *p* < 0.001) and then a rapid decline over time (*S* = −5.257, *p* = 0.007), referred to as the “low resilience-rapidly declining group.” The group named “low resilience-rapidly declining group” accounted for 12.13% of the sample (*n* = 29). Category 2: The caregivers in this group had a high level of family resilience initially (*I* = 92.469, *p* < 0.001) and then slowly increased over time(*S* = 2.502, *p* < 0.001) named “high resilience-slowly rising group.” The group named “high resilience-slowly rising group” accounted for 87.87% of the sample (*n* = 210). Error bars indicate the standard error (SE) at each time point, representing the variability within each group.

**Table 3:** GMM model fitting information for different categories (*n* = 239).

Model	AIC	BIC	ABIC	H	*p*-value		Proportion of each type of sample (%)
					LMR	BLRT	
1	5915.320	5943.132	5917.774	—	—	—	—
**2**	**5705.348**	**5743.589**	**5708.722**	**0.999**	**<0.001**	**<0.001**	**87.87/12.13**
3	5695.235	5743.906	5699.530	0.915	0.0867	<0.001	79.92/12.13/7.95
4	5680.117	5739.217	5685.331	0.931	0.4514	<0.001	79.50/9.62/8.37/2.51

Bold values indicate the optimal model selected based on a comprehensive evaluation of fit indices (AIC, BIC, ABIC, Entropy, LMR, BLRT), class proportions, and clinical interpretability.

### 3.3 Predictors of family resilience in caregivers with dementia

Univariate analysis indicated significant differences between the two trajectory categories across several variables. The Hosmer-Lemeshow test for the logistic regression model yielded a non-significant result (*χ*^2^ = 8.67, *df* = 8, *p* = 0.370), indicating good calibration. Factors with *p* < 0.05 included Disease Course, Self-rated Health, Relationship with the Patient, Daily care duration, Disease severity, Activities of daily living, Dementia knowledge, Social Support, Self-efficacy, and Caregiver empowerment (Table 2).

### 3.4 Prediction of family resilience trajectories based on a binary logistic regression model

The binary logistic regression model analysis showed that Relationship with the Patient, Self-rated Health, Dementia knowledge, Social Support, Self-efficacy, and Caregiver empowerment were predictors of potential categories of family resilience trajectories. See Table 4 for details.

**Table 4:** Binary logistic regression model analysis of latent categories of family resilience trajectories in dementia caregivers.

Variables	*β*	SE	Wald	*p*	OR	95% CI
Relationship with the patient						
Spouse					1.000	
Others	−1.936	1.142	2.873	0.090	0.144	0.015 ~ 1.353
Kid	−2.445	1.231	3.945	0.047	0.087	0.008 ~ 0.968
Self-rated health						
Poor					1.000	
Average	2.243	1.022	4.819	0.028	9.418	1.272 ~ 69.747
Good	3.990	1.900	4.410	0.036	54.040	1.305 ~ 2237.746
Daily care duration						
≥4 h and <8 h					1.000	
≥8 h and <12 h	−1.128	1.316	0.735	0.391	0.324	0.025 ~ 4.268
≥12 h	−0.979	1.286	0.580	0.446	0.376	0.030 ~ 4.670
Disease severity						
Mild					1.000	
Moderate	1.816	1.047	3.010	0.083	6.146	0.790 ~ 47.806
Severe	1.173	1.536	0.583	0.445	3.232	0.159 ~ 65.669
Activities of daily living						
Mild					1.000	
Moderate	−1.373	1.044	1.729	0.189	0.253	0.033 ~ 1.961
Severe	0.746	1.691	0.195	0.659	2.109	0.077 ~ 58.018
Dementia knowledge	0.193	0.075	6.604	0.010	1.213	1.047 ~ 1.406
Social support	0.236	0.098	5.819	0.016	1.266	1.045 ~ 1.534
Self-efficacy	0.126	0.068	3.474	0.062	1.134	0.994 ~ 1.295
Caregiver empowerment	0.755	0.197	14.639	<0.001	2.128	1.445 ~ 3.132
Disease course	−0.237	0.154	2.368	0.124	0.789	0.584 ~ 1.067
Constant	−29.652	7.637	15.077	<0.001	<0.001	

### 3.5 Prediction of family resilience trajectory from decision tree model

**Figure 3:**
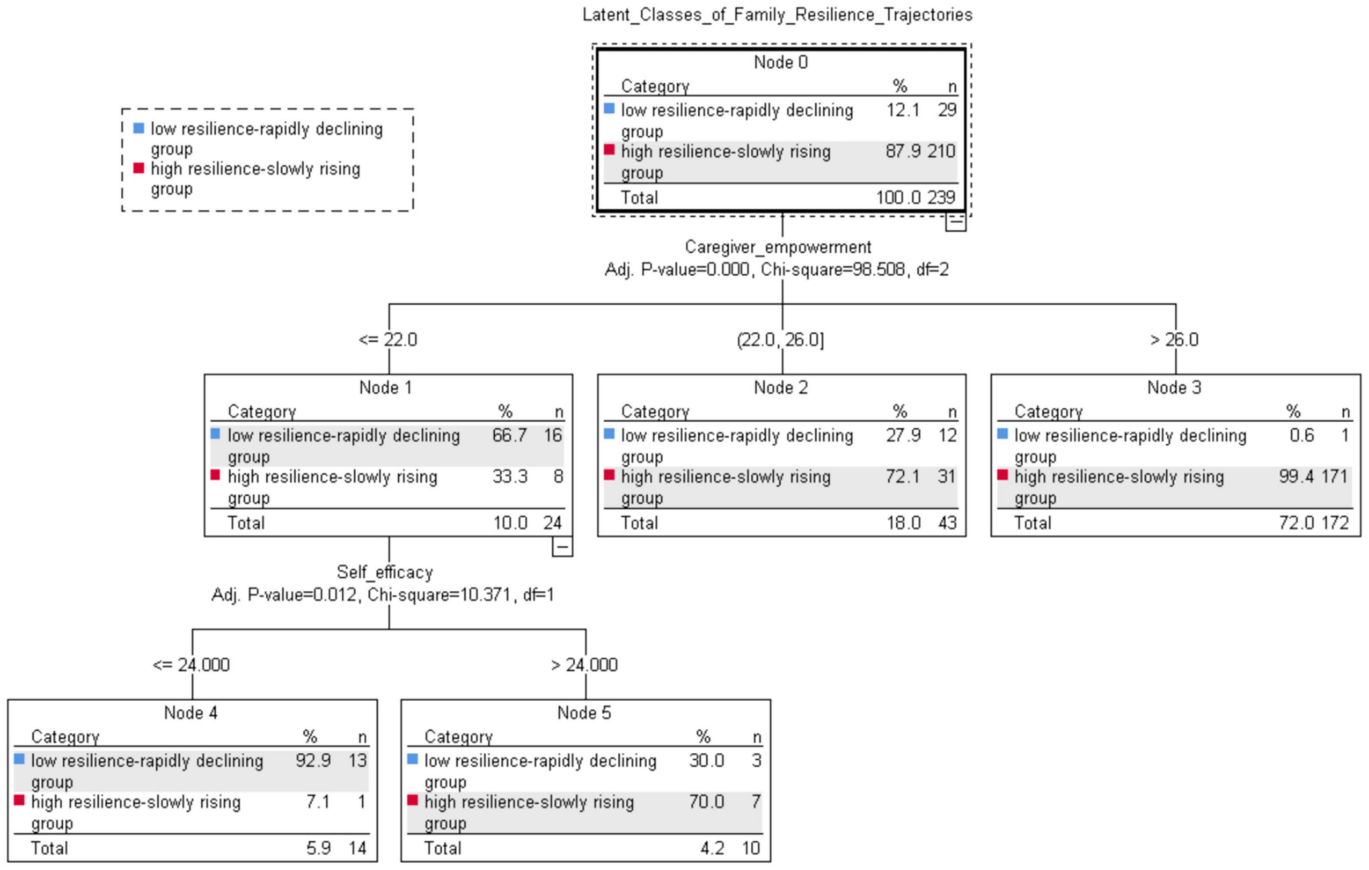
Family resilience trajectory decision tree model.

The resulting terminal decision tree model is illustrated in Figure 3. It comprises two layers of decision tree growth, consisting of a total of six nodes, including four terminal nodes. These nodes were screened to identify the two explanatory variables: Caregiver Empowerment and Self-efficacy. The first layer was Caregiver empowerment, suggesting that Caregiver empowerment was most relevant to Latent Classes of Family Resilience Trajectories. Caregivers of dementia with a Caregiver Empowerment score ≤22 had a 66.7% probability of falling into the low resilience-rapidly declining group. Conversely, those caring for patients with a score > 26 had a 99.4% probability of belonging to the high resilience-slowly rising group. In the subgroup of Caregiver Empowerment score ≤22, influenced by Self-efficacy score, the likelihood of caregivers of people with dementia with Self-efficacy score ≤ 24 belonging to the low resilience-rapidly declining group was 92.9%; the possibility that a caregiver of people with dementia with a Self-efficacy score >24 belonged to the high resilience-slowly rising group was 70.0%.

### 3.6 Performance between the logistic regression model and the decision tree analysis

**Figure 4:**
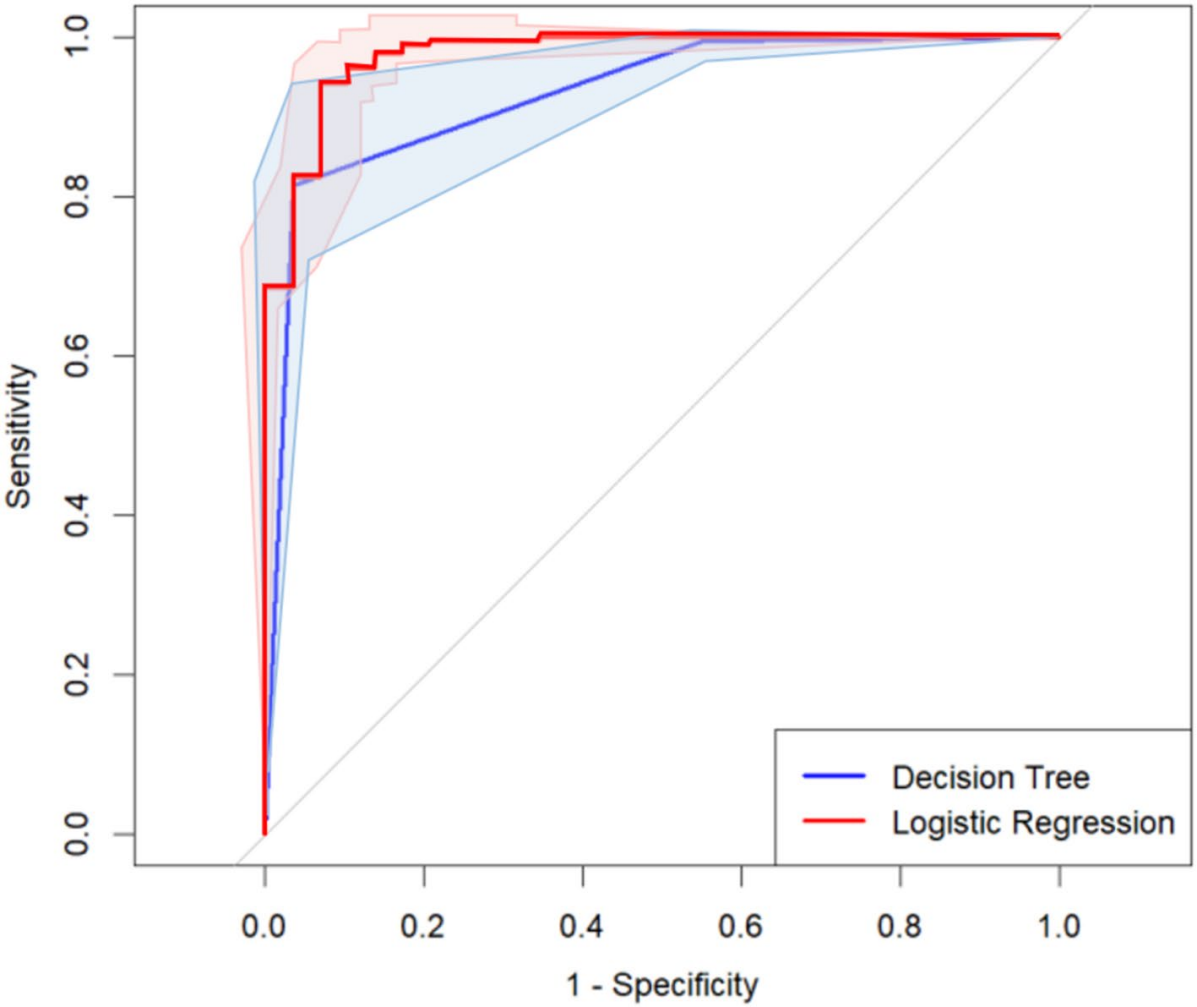
The ROC curves of the latent categories of family resilience trajectories of dementia caregivers in the logistic regression model and the Decision Tree model.

As shown in Figure 4 and Table 5, the *AUC* of the decision tree model for the potential categories of family resilience trajectories for dementia caregivers was 0.930, with a Youden index of 0.780, a sensitivity of 81.4%, and a specificity of 96.6%. The binary logistic regression model had an *AUC* of 0.978, with a Youden index of 0.874, a sensitivity of 94.3%, and a specificity of 93.1%. The shaded areas around the curves represent the 95% confidence bands, reflecting the stability and robustness of the predictive performance. The calibration plot for the decision tree model showed acceptable agreement between predicted and observed probabilities, though with some deviation in higher-risk groups due to limited sample size. The Delong test result is *Z =* 3.332, *p <* 0.001, indicating that the logistic regression model outperforms the Decision Tree model in evaluating its efficacy. The regression model has higher sensitivity, while the Decision Tree model has higher specificity.

**Table 5:** Parameters of ROC curves for potential categories of family resilience trajectories for dementia caregivers with the decision tree model and logistic regression model.

Model	AUC	SE	95% CI	Sensitivity	Specificity	Z	*p*
Decision tree	0.930	0.023	0.885 ~ 0.975	0.814	0.966	3.332	<0.001
logistic regression	0.978	0.013	0.951 ~ 1.000	0.943	0.931	—	—

## 4 Discussion

In this longitudinal study of Chinese dementia caregivers, we identified two distinct trajectories of family resilience: a “low resilience-rapidly declining” group and a “high resilience-slowly rising” group. Furthermore, we employed an integrated dual-model approach, combining logistic regression and decision tree analysis, to predict trajectory membership. These findings deepen our understanding of aging and caregiving research: First, the study found that adult children constitute the majority of primary caregivers. These findings highlight the evolving reality in which middle-aged and older adults are increasingly assuming informal caregiving responsibilities, which has multiple implications for their own health trajectories; Second, our longitudinal design captures the dynamic nature of caregiving resilience over time, underscoring the need for trajectory-based frameworks in future research; Third, the empowerment thresholds identified in the study provide candidate criteria for early risk stratification in community-based older adult care settings, which has direct implications for the design of tiered interventions and caregiver support policies in aging societies. In both models, caregiver empowerment emerged as a dominant predictor, suggesting that empowerment-focused interventions may offer an evidence-based strategy for preventing a decline in resilience among high-risk caregiving households. These results may provide an empirical basis for developing tiered, empowerment-focused interventions aimed at preventing resilience decline among high-risk caregiving families.

### 4.1 Caregiver empowerment as the strongest predictor

Caregiver empowerment was found to be a common predictor of both models. Caregivers with higher caregiver empowerment scores reported better professional competence, cognitive mindfulness, physical and mental state, and external support. Good professional practice skills can enhance caregiving professionalism. They reduce uncertainty in coping with the disease, decrease caregiving errors and family anxiety, and create a stable family environment (35). This finding aligns with Hogstedt’s study, which showed that caregivers’ structural empowerment is positively associated with professional competence and self-assessment (36). Understanding the nature of dementia caregiving helps caregivers adjust unreasonable expectations. It also establishes a sense of mission, maintains family harmony with a rational and positive mindset, and enhances family cohesion when facing difficulties (37). Taking care of oneself and the patient protects the caregiver’s physical and mental health, preventing fatigue-related family conflicts. At the same time, it reduces the patient’s behavioral symptoms and lowers the overall family burden by optimizing care quality (23). These benefits are consistent with the concept of psychological empowerment proposed by Yu (38). Previous studies have shown that peer support provides emotional comfort and shared experience, relieving psychological stress and broadening coping strategies. Collaboration between caregivers and professionals also strengthens psychological resilience and adaptive capacity (39). In summary, our results suggest that caregiver empowerment enhances the caregiver’s ability to manage stress across four dimensions: professional skills, cognitive understanding, physical and mental well-being, and external resources. By building competence in each area, it improves the family’s overall adaptability, stability, and resilience when confronting the demands of dementia care. These findings have direct implications for intervention design: caregiver training programs should adopt a multi-dimensional empowerment framework that addresses professional skills, psychoeducation, self-care, and peer networking, thereby maximizing the protective effect on family resilience.

### 4.2 The role of self-efficacy and its interaction with empowerment

Logistic regression results showed that self-efficacy showed a positive trend in predicting caregivers’ family resilience development trajectories after controlling for other variables. Dementia caregivers with high self-efficacy are typically more optimistic in their own caregiving abilities and adopt proactive coping strategies rather than passive avoidance in the face of stressful events during the caregiving process. Additionally, Hussien (40) study demonstrated that high self-efficacy enabled caregivers to rationalize caregiving frustrations and reduce self-denial and emotional exhaustion. However, its marginal significance suggests that the effect of self-efficacy may be relatively weak, potentially influenced by multiple predictors, or might vary across different subgroups. Decision tree modeling clarified this interaction: the influence of self-efficacy on resilience trajectories was contingent on the level of caregiver empowerment. Among high-empowerment caregivers, whose trajectories were generally characterized as “high resilience-slowly rising,” the additional influence of self-efficacy was attenuated. In contrast, among low-empowerment caregivers, self-efficacy played a critical differentiating role: up to 92.9% of those with both low empowerment and low self-efficacy belonged to the “low resilience-rapidly declining” group. According to Bandura’s theory of self-efficacy (41), an individual’s belief in their own abilities drives their behavior and persistence. Among caregivers of people with dementia, low levels of empowerment often indicate a lack of practical skills and cognitive understanding. When these deficiencies are combined with low self-efficacy, the situation worsens further. Ultimately, family members may begin to question the “quality of care,” thereby exacerbating conflicts over caregiving responsibilities and accelerating the decline in family resilience. These findings suggest that healthcare systems could consider routine screening using the identified caregiver empowerment threshold to detect high-risk families. However, directly recommending training for the low-resilience subgroup is premature, as these caregivers may have cognitive impairments that affect intervention feasibility. Further research is needed to assess their specific needs and the appropriateness of self-efficacy training before any program can be integrated into community dementia care.

### 4.3 Health status, dementia knowledge, and relationship with the patient

The physical and mental health of caregivers is a crucial foundation for developing resilience (42). In the present study, caregivers who rated their own health as “Good” or “Average” were more likely to be classified into the “high resilience-slowly rising” group, whereas those who rated their health as “Poor” were more likely to be classified into the “low resilience-rapidly declining” group. This may be because good physical functioning enables caregivers to maintain their daily caregiving activities, while a good mental health status helps them deal effectively with emotional stress (43). These basic health resources become a crucial support for maintaining good resilience when caregivers face stress. This is consistent with Wendlandt (44) finding that caregivers with low psychological resilience and self-rated health are more likely to have a developmental trajectory of persistently low chronic posttraumatic stress symptoms (PTSS). This suggests that any intervention aimed at enhancing caregiver resilience should focus on their basic health needs first.

In addition to health as a foundation, caregivers’ dementia-specific knowledge, that is, their understanding of the disease course, symptom management, and appropriate caregiving strategies as measured by the DKAS, has a direct impact on their caregiving abilities, patient outcomes, and their own mental health (45). In this study, we found that the advantage ratio of belonging to the “high resilience-slowly rising group” increased by 1.2 times for every unit increase in the caregiver’s Dementia knowledge. Caregivers with high dementia knowledge can anticipate the evolution of the disease and reduce the fear and anxiety caused by a lack of knowledge. This knowledge can be translated into effective caregiving, improving the quality of care for patients with dementia (46). However, Pokala (47) study concluded that higher Dementia knowledge did not reduce caregivers’ caregiving burden, which may be due to the study’s consideration of the role of socio-economic conditions, where higher Dementia knowledge reinforces caregivers’ perceived responsibility and behavioral demands for professional caregiving. Lower incomes prevent caregivers from purchasing necessary tools, medications, hiring helpers, and other resources to alleviate caregiving stress, resulting in a disproportionate concentration of family caregiving responsibilities on themselves and ultimately increasing their caregiving burden.

In the relationship with the patient, caregivers were at a higher risk of belonging to the “low resilience-rapidly declining group” compared to spouses, which is consistent with the findings of Fenton (48). The reason for this may be that adult children have difficulty in balancing family and professional responsibilities due to work and family situations. It may show higher socio-emotional and economic burdens. In contrast, spouses have a long-term relationship with people with dementia, which allows them to draw energy from the deep affection in the face of caregiving dilemmas and to cope with the challenges in a positive frame of mind, thereby contributing to the maintenance and enhancement of the family’s resilience (49). As a result, clinical and community service agencies can offer programs such as weekend respite care, financial assistance, and psychoeducation to provide additional support to adult-child caregivers, thereby maintaining family resilience and improving the quality of dementia care.

### 4.4 Social support as an external protective factor

This study found that social support, as an external protective factor, plays a significant role in maintaining family resilience. Social support operates at two distinct levels. At the individual level, it encompasses emotional support, life satisfaction, and optimism. At the environmental level, it encompasses social capital and collective efficacy, both of which facilitate the integration of caregivers into broader social networks. These networks foster the exchange of emotions and practical information, creating a mutually reinforcing cycle of support that improves family resilience (50). The study by Salinas also emphasized the link between a broader social support network and higher cognitive resilience (51), which aligns with the present study’s finding that caregivers with higher social support exhibit stronger family resilience. These findings carry concrete implications for practice and policy. Healthcare providers should incorporate social support assessments into routine caregiver evaluations, enabling early identification of socially isolated caregivers at elevated risk. At the policy level, governments and community organizations should collaborate to establish structured support networks for dementia caregivers, including peer support groups, community health worker programs, and respite care services, as integral components of national dementia care strategies.

### 4.5 Comparison of logistic regression and decision tree models

The reason that the statistically significant variables Relationship with the Patient, Self-rated Health, Dementia knowledge, and Social Support in logistic regression did not enter the decision tree model may stem from the essential difference in the variable selection mechanism between the two models. Logistic regression can identify statistically significant predictors, even if their effect sizes are relatively weak, by assessing the independent net effect of each dependent variable after controlling for other variables. However, the decision tree model employs a recursive partitioning algorithm with a greedy strategy to prioritize variables that provide the most significant immediate segmentation efficacy. Those variables that are statistically significant but have relatively weak predictive strengths or interact with other strong predictors are mistakenly eliminated as confounders during the construction of the decision tree, resulting in their failure to be selected for the final model. In addition, this study demonstrated the superior assessment efficacy of the logistic regression model by comparing the AUC results. Still, in general, the logistic regression model demonstrated higher sensitivity and effectively identified potential predictors related to the outcome. In contrast, the decision tree model demonstrated higher specificity, focusing on screening out core variables with strong discriminatory ability. The combination of the two types of models can help to integrate their methodological advantages, which not only improves the predictive efficacy but also systematically reveals the key predictors affecting the potential categories of family resilience trajectories of caregivers with dementia, thus providing a more comprehensive and in-depth theoretical basis for the development of more targeted intervention strategies.

Rather than being directly combined into a unified ensemble model, the two approaches were applied in parallel to provide complementary perspectives. Logistic regression provided estimates of independent predictor effects with associated uncertainty quantification, while the decision tree generated interpretable hierarchical decision rules that may inform clinical screening logic. The convergence of both approaches in identifying caregiver empowerment as the dominant predictor strengthens confidence in this finding. However, we do not claim that combining the two models improves predictive accuracy in any formal ensemble sense; future work could explore stacked or weighted ensemble approaches with appropriate cross-validation.

### 4.6 Family resilience trajectories in the context of healthy aging

The two resilience trajectories identified in this study may offer broader insights into the aging process of caregivers themselves. First, it is worth noting that a significant proportion of caregivers belong to the middle-aged and older adult population; this demographic characteristic underscores a clinically meaningful overlap between caregiving demands and the health challenges of later life, a connection that warrants prospective investigation (52). Conceptually, the predictive factors distinguishing these two trajectory groups align with the resources previously associated with healthy aging outcomes in longitudinal studies. Caregivers in the “high resilience-slowly rising” group exhibited significantly higher levels of empowerment, self-efficacy, knowledge of dementia, and social support. Existing evidence preliminarily suggests that these factors are associated with the maintenance of physical function in later life, reduced burden of depressive symptoms, and sustained social participation (11, 53). Whether the accumulation of these resources over the observation period translates into measurable benefits for healthy aging remains an empirical question that requires validation through prospective studies. Conversely, caregivers in the “low resilience-rapid decline” group may, due to their resource status, be more susceptible to stress-related health deterioration over time; previous studies have shown that sustained caregiving stress under resource-poor conditions is associated with increased compensatory burden among older caregivers and heightened risks of depression and cognitive sequelae (52). Whether resilience trajectories precede or follow these health changes is a question that longitudinal designs with objective health measures are better positioned to address. The social dimensions of healthy aging warrant further consideration. The low-resilience group’s lower baseline levels of social support, combined with markedly high caregiving intensity, suggest a pre-existing vulnerability that may limit their social participation over time (11, 52); given the 18-month observation window of the present study, whether this vulnerability intensifies or attenuates beyond the follow-up period remains an important question for future research. Taken together, these findings suggest that empowering caregivers may not only serve as a protective factor for family resilience but also represent a potential entry point for supporting caregivers in maintaining their own health and social participation as they age (54). Future longitudinal studies are needed to directly measure caregivers’ health outcomes across physiological, psychological, and social domains to test these hypotheses and determine whether resilience trajectories hold independent predictive value for healthy aging in this population.

## 5 Limitations and future directions

This study has several limitations that should be taken into consideration. First, the use of convenience sampling limits the generalizability of the findings, particularly to caregivers outside of Zhejiang Province. Furthermore, it should be noted that only one patient died during the 1.5-year follow-up period. This low mortality rate may suggest that the sample predominantly consisted of individuals in the early to moderate stages of dementia, which could limit the generalizability of the findings to populations with advanced dementia or higher mortality risk. Future research should aim for a more diverse sample, including caregivers of patients across the full spectrum of dementia severity, to enhance the external validity of the results. Second, although our total sample size met the *a priori* requirement for Growth Mixture Modeling derived from Kim (26) simulation study, the absolute number of caregivers classified into the “low resilience-rapidly declining” subgroup was small, and the imbalanced distribution between the two trajectory groups may have affected the statistical power and stability of our predictive models, particularly with respect to that subgroup. This imbalance could also potentially lead to overfitting in the decision tree model. Future studies with larger and more balanced samples, especially a larger proportion of low-resilience caregivers, are needed to confirm the thresholds identified here. Third, all variables were assessed through self-reported questionnaires, which are susceptible to recall bias, social desirability bias, and the influence of caregivers’ emotional states at the time of assessment. Furthermore, the mixed-mode data collection approach, combining face-to-face, telephone, and WeChat-assisted interviews across time points, may have introduced additional interviewer effects and response inconsistencies. Fourth, the present study employed a “class assignment” approach for subsequent predictive analyses, where each caregiver was assigned to their most likely trajectory class. This method does not account for the uncertainty inherent in latent class membership, which may lead to an underestimation of standard errors and an increased risk of Type I errors in the logistic regression and decision tree models. Future studies should consider incorporating objective indicators, such as physiological stress markers or standardized clinical assessments, to complement self-reported data and improve measurement validity.

## 6 Conclusion

This longitudinal study identified two distinct family resilience trajectories among Chinese dementia caregivers, “low resilience-rapidly declining” and “high resilience-slowly rising,” using Growth Mixture Modeling, with complementary logistic regression and decision tree analyses to identify trajectory predictors. Caregiver empowerment emerged as the dominant predictor across both models, with self-efficacy, self-rated health, dementia knowledge, social support, and caregiver, patient relationship also contributing. These findings demonstrate that family resilience is a dynamic, modifiable process shaped by internal and external resources, and that middle-aged and older adults increasingly bear the informal caregiving burden in aging societies. The empowerment-based thresholds identified by the decision tree offer candidate criteria for early identification of high-risk families, pending external validation. For practice and policy, we recommend integrating routine resilience and empowerment screening into community-based geriatric care, and developing tiered, empowerment-focused interventions that prioritize intensive support for vulnerable caregiving families, thereby preventing resilience decline and improving quality of care for older adults with dementia.

## Data Availability

The datasets presented in this article are not readily available because the datasets generated and analyzed during the current study are not publicly available due to ethical and privacy restrictions. The data contain sensitive personal information about family caregivers and older adults with dementia, including health status, family relationships, and psychological assessments. According to the terms of ethical approval granted by the Ethics Committee of Huzhou University (No. 202401–17) and the informed consent obtained from participants, the data are restricted to use only for the purposes described in this study. However, anonymized data may be available from the corresponding author upon reasonable request and with appropriate ethical approval for secondary analysis purposes. Requests to access the datasets should be directed to JL, 18235473006@163.com.

## Glossary

BPSD: Behavioral and psychological symptoms of dementia
ADL: Activities of daily living
IADL: Instrumental activities of daily living
FRAS: Family resilience assessment scale
EFCD: Empowerment scale for family caregivers of community-dwelling people with dementia
SSRS: Social support rating scale
DKAS: Dementia knowledge assessment scale
GMM: Growth mixture modeling
I: Intercept
S: Slope
AIC: Akaike information criterion
BIC: Bayesian information criterion
ABIC: Adjusted bayesian information criterion
H: Entropy
LMR: Lo–Mendell–Rubin
BLRT: Bootstrap-based Bootstrap Likelihood Ratio Test
BLRM: Binary logistic regression model
CHAID: Chi-square automatic interaction detection
ROC: Receiver operating characteristic
AUC: Area under the curve
